# Role of IL-17A and IL-10 in the antigen induced inflammation model by *Mycoplasma pneumoniae*

**DOI:** 10.1186/1471-2180-14-156

**Published:** 2014-06-13

**Authors:** Satoshi Kurata, Takako Osaki, Hideo Yonezawa, Ken Arae, Haruhiko Taguchi, Shigeru Kamiya

**Affiliations:** 1 Department of Infectious Diseases, Kyorin University School of Medicine, Shinkawa 6-20-2, Mitaka, Tokyo 181-8611, Japan; 2 Department of Immunology, Faculty of Health Sciences, Kyorin University, Miyashitacho 476, Hachioji, Tokyo 192-8508, Japan

**Keywords:** *Mycoplasma pneumoniae*, Th17, Animal models, Immune response, Cytokines

## Abstract

**Background:**

*Mycoplasma pneumoniae* is one of the causative organisms of community-acquired pneumonia which is found commonly in younger patients. Extrapulmonary complications similar to autoimmune disease are caused by *M. pneumoniae* following the initial infection. The mechanism and pathology of onset is not clear, but it is considered that excessive host immunoreactions play a part in the onset of mycoplasmal pneumonia and its extrapulmonary complications. In this study, we investigated the participation of the immune response, excluding the participation of Th1 and Th2 which has previously been investigated.

**Results:**

In this study, the host immune response of an antigen induced inflammation model using SPF mice repeatedly sensitized with *M. pneumoniae* antigens was analyzed. The specificity of *M. pneumoniae* antigens in the Th17 response of murine lymphocytes in vitro was also examined. Frequent and concentrated sensitization induced exacerbation of lung inflammation immunologically and pathologically, and evoked intrapulmonary IL-17A and IL-10 production. *M. pneumoniae* antigen stimulation induced proliferation of mouse lymphocytes and caused production of IL-17A and IL-10. In addition, it was shown that IL-17A and IL-10 production was increased in the presence of IL-6 and TGF-β1.

**Conclusions:**

It was shown that *M. pneumoniae* antigens induced potent immunoreaction and enhanced the Th17 cell response both in vivo and in vitro, and that both Treg and IL-10 are involved in the suppression of IL-17A production. This raises the possibility that breakdown of the immune balance may be part of the process leading to subsequent development of extrapulmonary mycoplasmal pneumonia.

## Background

Mycoplasmas are the smallest bacteria capable of autonomous replication, and these microorganisms are unique in that they lack a bacterial cell wall. *M. pneumoniae* is an etiologic agent responsible for community-acquired respiratory tract infections (primary atypical pneumonia, PAP) mainly in school-age children and young adults. *M. pneumoniae* can spread from person to person via droplets, attaching to human airway epithelial cells via the P1 protein, one of the tip components of an adherent organ on the bacterial cell surface [1,2].

Recently, it has been reported that the community-acquired respiratory distress syndrome toxin (CARDS Tx) which possesses adenosine diphosphate-ribosyltransferase activity similar to *Bordetella pertussis* toxin is produced by *M. pneumoniae*[3]. CARDS Tx was not secreted into the culture supernatant, but localized to the cytoplasmic and cell membranes, inducing vacuolating cytotoxicity. However, it is difficult to explain the pathogenic mechanisms of mycoplasmal pneumonia in relation to *M. pneumoniae* virulence factors. Clinical symptoms of mycoplasmal pneumonia in early childhood are not marked and manifestations of *M. pneumoniae* infection such as pneumonia appear only in school-age or older children [4]. Severe inflammatory responses in the lung are also not commonly observed in *M. pneumoniae* infected immunocompromised hosts [5]. According to the report by Tanaka et al. [6], it was inferred that accumulation of lymphocytes and monocytes activated by *M. pneumoniae* infection in the bronchi and lung tissue leads to both insufficiency of lymphocytes at the periphery and negative conversion in the tuberculin test. Furthermore, it was reported that the onset of various autoimmune type extrapulmonary complications such as Guillain-Barré syndrome, Stevens-Johnson syndrome, hepatitis, myocarditis and arthritis were observed subsequent to *M. pneumoniae* infections [7-10]. Consequently, the participation of the excessive host immune response is thought to be involved in the severity of mycoplasmal pneumonia and also the onset of complications [11,12].

In recent years, a third positive effector T cell subset known as Th17 cells were characterized by abundant production of IL-17 [13,14]. IL-17 is more important than IFN-γ in onset and exacerbation of autoimmune diseases such as collagen-induced arthritis (CIA) and experimental allergic encephalitis (EAE), which are thought to be pathogenetically induced by the Th1 immune response [15,16].

On the other hand, inducible regulatory T cells (iTreg) such as Tr1 and Th3 have been reported to contribute to the suppression of the hyperimmune response [17,18].

It was reported that the Th17 cells are induced by segmented filamentous bacteria (SFB) which colonize the intestinal tract [19]. However, the relationship of Th17 cells with the pathogenic mechanisms of mycoplasmal pneumonia and its extrapulmonary complications are not clear. Treg has not previously been identified as an inhibiting factor of the *M. pneumoniae* inflammatory response.

We have previously reported that experimental pneumonia can be caused by intranasal inoculation of *M. pneumoniae* soluble sonicated antigens to specific pathogen-free (SPF) mice [20,21]. In the present study, we prepared a *M. pneumoniae* antigen induced inflammation model by use of SPF mice recurrently inoculated with *M. pneumoniae* antigens and performed pathological and immunological analyses to examine the induction mechanisms of Th17 and Treg cells. Additionally, we investigated the specificity of Th17 and Treg cell inducibility with mouse lymphocytes in vitro by using various bacterial antigens and immunoactivatory components.

## Methods

### Bacterial strains and culture conditions

The reference strain *M. pneumoniae* M129, stocked at the Department of Infectious Diseases, Kyorin University School of Medicine was used in this study.

*M. pneumoniae* cells were cultured at 37°C under a 5% CO_2_ atmosphere for 7 days in PPLO broth (Oxoid, Hampshire, UK) containing mycoplasma supplement-G (Oxoid) for the preparation of soluble *M. pneumoniae* antigens. *Klebsiella pneumoniae* (ATCC 13883; American Type Culture Collection, Rockville, MD) and *Streptococcus pneumoniae* (ATCC 33400) were cultured at 37°C under aerobic conditions for 18 hours in brain heart infusion broth (BHI; Becton Dickinson, MD) (BD Difco Franklin Lakes, NJ).

### Experimental animals and breeding conditions

Specific pathogen-free (SPF) female BALB/cAJcl mice (5 weeks of age for antigen induced inflammation model, and 6 weeks of age for harvest of splenocytes) were purchased from Clea Japan (Tokyo, Japan) and bred in isolators.

### Preparation of sonicated *M. pneumoniae* crude antigens

*M. pneumoniae* soluble antigens were prepared as previously described [20,21]. The cultured bacteria were harvested and washed 5 times by centrifugation at 10000 × g for 20 min (*M. pneumoniae*) or 3000 × g for 15 min (*K. pneumoniae* and *S. pneumoniae*) in Hanks’ balanced salt solution (Gibco, New York, USA). The cells were suspended in saline and sonicated 10 times for 1 min per burst at output 7 (Sonifier 250, Branson Ultrasonic Corporation, Danbury, CT, USA). The supernatant was decanted after centrifugation at 10000 × g for 5 min, and served as crude soluble antigen. The protein concentration of the suspension was measured using the Bio-Rad Protein Assay (Hercules, CA, USA).

### Inoculation and sensitization conditions

Animal experiments were approved by the Institutional Animal Care and Use Committee of Kyorin University School of Medicine (Approval No. 95, 95–1, 95–2).

Mice were anaesthetized intraperitoneally with 25 mg/kg body weight of sodium pentobarbital (Dainippon Sumitomo Pharma, Osaka, Japan). SPF mice in Group A were intranasally inoculated once a week for 5 weeks with sonicated crude antigens prepared from *M. pneumoniae* strain M129 (1 mg protein/kg/5 times). The inoculated protein doses were changed in Groups B and C. In Group B, lower doses (0.1 mg/kg) of the antigen were inoculated once a week at day 0, 7 and 14, and higher doses (1 mg/kg) of the antigen were used for the last inoculation at day 28. In Group C, crude antigen (1 mg/kg) was inoculated at day 0 and 28 only. Control mice in Group D were inoculated with saline once a week for 5 weeks (n = 5 or 6 in each group).

### Pathological examination

Mice were sacrificed on the day after the last sensitization. The intermediate and lower lobes of the right lungs of the mice were fixed in 5% formalin. Sections of paraffin-embedded tissues were stained with hematoxylin and eosin and analyzed by light microscopy.

### Intrapulmonary mRNA gene expression analysis

Total RNA was extracted from the upper lobe of the right lungs of the mice using the QIAzol, QIAshredder and RNeasy Mini spin column RNA isolation Kit (QIAGEN GmbH, Hilden, Germany). cDNA was synthesized from sample RNA using ReverTra Ace RT PCR Kit (TOYOBO CO., LTD, Osaka, Japan). All real-time PCRs were performed with SYBR Green Premix Ex Taq (TaKaRa Bio Inc., Shiga, Japan) by the ABI 7500 Fast Real-Time PCR System (Applied Biosystems, Inc. Carlsbad, California, US) as described previously [22-25] using specific primers for individual genes. Fold changes of targeted genes of each sample were relatively quantified using threshold cycle (Ct) values and calculated using the ddCT method normalizing B-actin or 18S RNA values.

### In vitro analysis for specificity of differentiation inducing activity of Th17 cells by *M. pneumoniae* antigens

The spleens were removed from three mice for each experiment. Lymphocytes were separated from the spleens of BALB/c mice by Lympholyte M (Cedarlane Laboratories Limited, Hornby, Ontario, Canada). Lymphocytes (8 × 10^4^ cells/0.2 ml) were then incubated with 20 ng/ml of mouse IL-6 (R&D Systems, Minneapolis, MN, USA) plus 2 ng/ml of human TGF-β1(R&D Systems) at 37°C under 5% CO_2_ for 4 days in RPMI 1640 medium (Invitrogen, Carlsbad, CA) supplemented with 10% fetal calf serum (FCS; Gibco), 10 μM 2-mercaptoethanol (MP Biomedicals, Fountain Parkway, Solon, OH), 50 μg/ml gentamicin (Schering Plough, Osaka, Japan) and 2.5 μg/ml amphotericin B (Bristol-Myers Squibb, Tokyo, Japan) [26].

In addition, lymphocytes were stimulated with the Dynabeads Mouse CD3/CD28 T Cell Expander (Invitrogen, Carlsbad, CA) during the incubation period. The sonicated crude antigens from *M. pneumoniae* strain M129, *K. pneumoniae* ATCC 13883, *S. pneumoniae* ATCC 33400, lipopolysaccharide from *Escherichia coli* O127:B7 (SIGMA-ALDRICH, St. Louis, MO, USA), and zymosan A from *Saccharomyces cerevisiae* (SIGMA-ALDRICH) were added to the culture. A culture without the addition of IL-6, TGF-β1 or antigens was included as control.

After 4-day culture, cell viability, based on mitochondrial succinic dehydrogenase activity was measured using a Cell Counting Kit-8 (Dojindo Molecular Technologies, Inc., Kumamoto, Japan) consisting of a WST-8 assay (2-{2-methoxy-4-nitrophenyl}-3-{4-nitrophenyl}-5-{2, 4-disulfophenyl}-2H-tetrazolium, monosodium salt). Culture supernatants were also harvested and assayed for cytokine activities by ELISA.

### Statistical analysis

Statistical evaluations were performed with Dunnett multiple comparison statistical test and Student’s *t*-test for comparisons between groups.

A value of p < 0.05 was considered to be statistically significant. Data are expressed as the mean ± the standard deviation.

## Results

### Histopathological analysis

**Figure 1:**
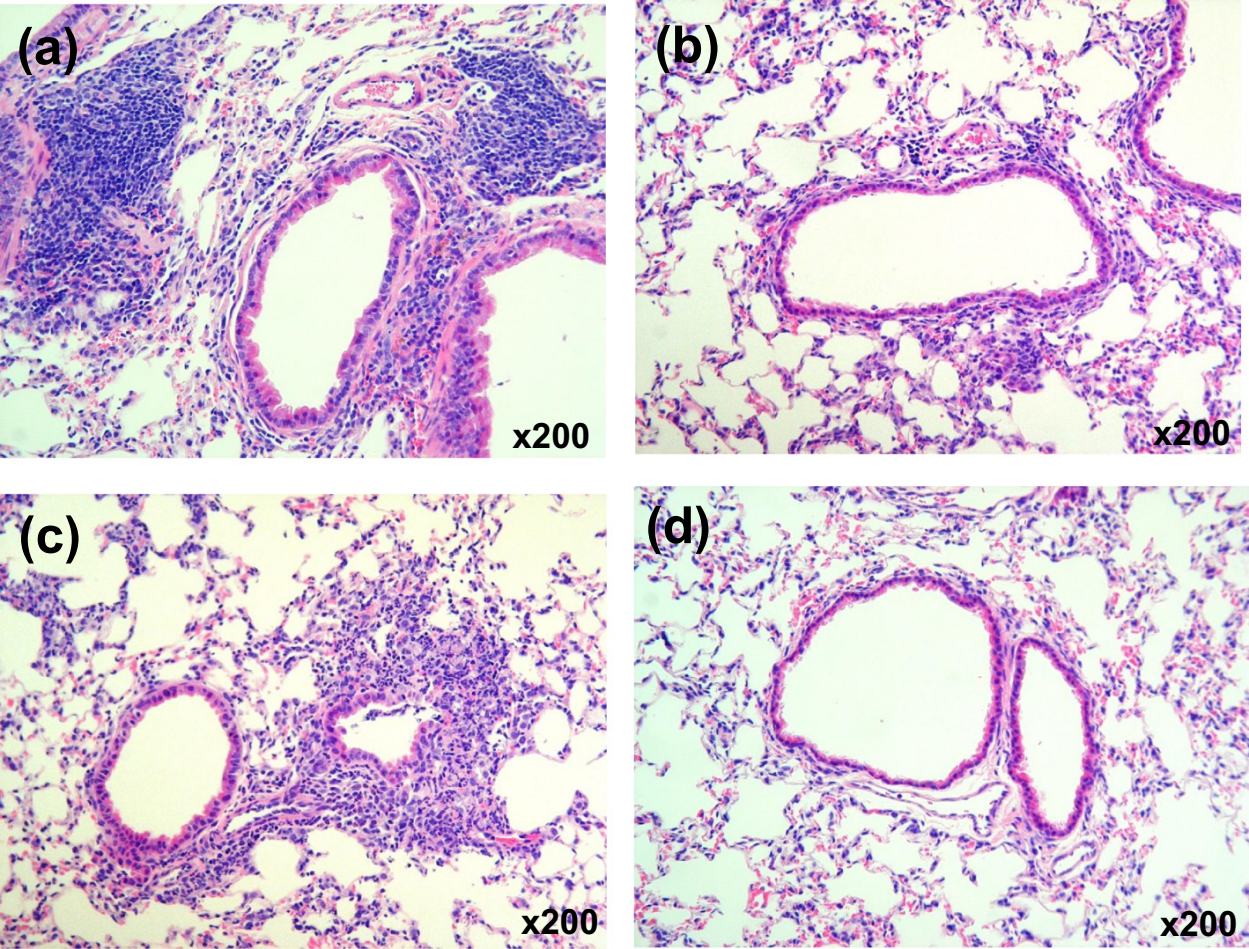
**Histopathology of the lung of BALB/c mice after intranasal sensitization with *M. pneumoniae*-sonicated antigens.** The figure shows hematoxylin and eosin staining of lung sections from mice repeatedly inoculated with *M. pneumoniae* antigens (day 29). Lung tissue sections from **(a)** Group A, **(b)** Group B, **(c)** Group C and **(d)** Group D (control) (magnification: × 200).

High dose and frequent *M. pneumoniae* antigen sensitization caused severe inflammatory changes including neutrophil infiltration and bronchial wall thickening in the lung tissues of Group A mice (Figure 1a). Low dose and frequent sensitization also induced neutrophilic infiltration in the lungs of the mice in Group B, but this inflammation was milder than that in Group A (Figure 1b). In Group C mice with high dose and infrequent sensitization, the inflammatory levels differed according to lung site and localized inflammation with neutrophil infiltration was observed (Figure 1c). No inflammatory cell infiltration was observed in any of the tissues in the saline control Group D mice (Figure 1d). These results demonstrated that high dose and frequent *M. pneumoniae* antigen sensitization induce significant inflammation in the lung.

### Immunological analysis for intrapulmonary cytokine protein quantification

**Figure 2:**
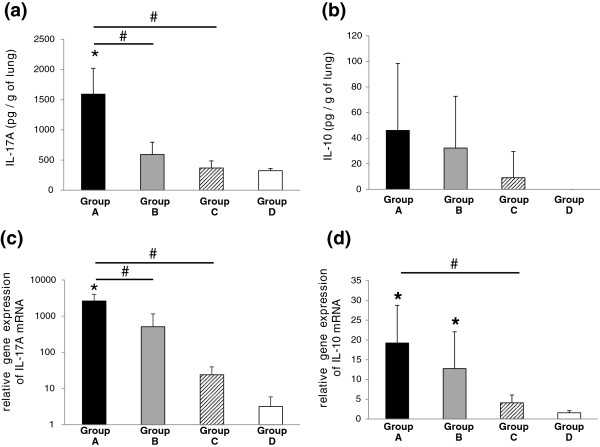
**Cytokine levels and relative quantification of cytokine mRNA levels in lung tissues of BALB/c mice. (a)** IL-17A levels per gram of lung tissue. **(b)** IL-10 levels per gram of lung tissue. **(c)** Relative quantification of IL-17A mRNA levels. **(d)** Relative quantification of IL-10 mRNA levels. Black bars, Group A mice; Grey bars, Group B mice; hatched bars, Group C mice; white bars, Group D mice. *p < 0.05, inoculate vs. Group D (control) by Dunnett multiple comparison statistical test, ^#^*p* < 0.05 by Student’s *t*-test.

In Group A mice, IL-17A levels in lung tissues were markedly increased (Figure 2a). Sensitization by lower doses of *M. pneumoniae* antigens also led to a rise in IL-17A levels in Group B mice. However, no significant changes were found in Group C mice. The levels of intrapulmonary IFN-γ and IL-4 in all mice were undetectable by ELISA (data not shown).

Intrapulmonary IL-10 production was not detected in control Group D mice, but sensitization with *M. pneumoniae* antigens induced the production of IL-10 in Groups A, B and C (Figure 2b).

**Figure 3:**
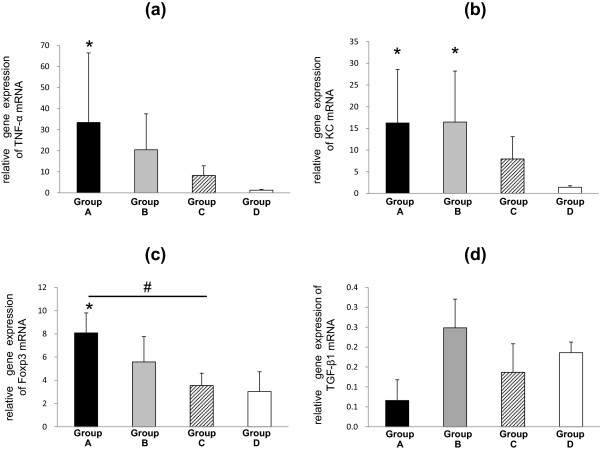
**Relative quantification of cytokine mRNA levels in lung tissues of BALB/c mice. (a)** Relative quantification of TNF-α mRNA levels. **(b)** Relative quantification of KC mRNA levels. **(c)** Relative quantification of Foxp3 mRNA levels. **(d)** Relative quantification of TGF-β1 mRNA levels. Black bars, Group A mice; Grey bars, Group B mice; hatched bars, Group C mice; white bars, Group D mice. *p < 0.05, inoculate vs. Group D (control) by Dunnett multiple comparison statistical test, ^#^*p* < 0.05 by Student’s *t*-test.

Statistically significant increases in IL-17A and IL-10 mRNA expression were shown to depend on frequency of sensitization and concentration of *M. pneumoniae* antigens used (Figure 2c,d). Relative quantification of tumor necrosis factor (TNF)-α mRNA and Keratinocyte-derived chemokine (KC) mRNA expression as an index of lung inflammation is shown in Figure 3a and b. Up-regulation of TNF-α mRNA and KC mRNA was observed in Groups A, B and C mice as expected according to histopathological findings. Forkhead box p3 (Foxp3) is a master regulator of CD4^+^CD25^+^ naturally occurring regulatory T cells (nTreg). Foxp3 mRNA was highly expressed in only Group A mice (Figure 3c). In contrast, no significant effect of *M. pneumoniae* antigens on TGF-β1 mRNA expression was observed in the lung (Figure 3d).

### In vitro analysis for specificity of differentiation inducing activity of Th17 cells by *M. pneumoniae* antigens

**Figure 4:**
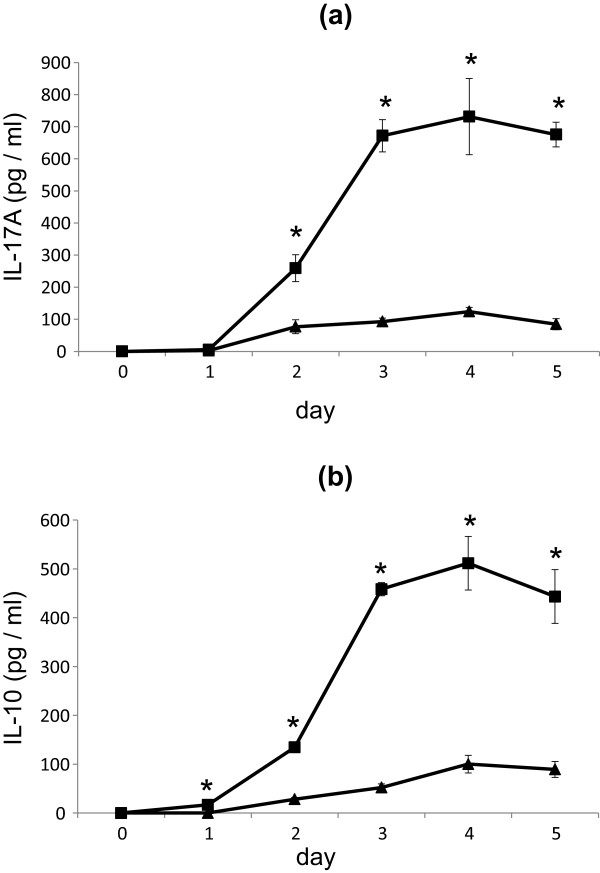
**Effects of *M. pneumoniae *antigens on cytokine production by murine lymphocytes.** Lymphocyte culture supernatant concentrations of **(a)** IL-17A (pg/ml), **(b)** IL-10 (pg/ml). Closed squares (■) show stimulation with 50 μg protein/ml of *M. pneumoniae* antigen. Closed triangles (▲) show saline control. *p < 0.05 vs. saline control by Student’s *t*-test.

Chronological cytokine production by *M. pneumoniae* antigens was examined. Lymphocytes were cultured with 50 μg protein/ml of *M. pneumoniae* antigens in the presence of IL-6 and TGF-β1. IL-17A concentration in the culture media was elevated from day 1 to day 4 and maintained at 600–700 pg/ml (Figure 4a). IL-10 production induced by *M. pneumoniae* antigens was observed to be maintained at 400–500 pg/ml (Figure 4b). In the saline control, elevation of IL-17A and IL-10 concentrations was up to 100 pg/ml at day 4 (Figure 4a,b).

### Effects of *M. pneumoniae* and other bacterial antigens on lymphocyte growth

Without IL-6 and TGF-β1, only 50 μg protein/ml of *M. pneumoniae* antigens promoted the proliferation of lymphocytes (Table 1). In the presence of IL-6 and TGF-β1, proliferation of lymphocytes was increased by either 10 or 50 μg protein/ml of *M. pneumoniae* antigens, while 50 μg protein/ml of either *S. pneumoniae* or *K. pneumoniae* sonicated antigens markedly decreased viable lymphocyte count. Similarly, in the presence of IL-6 and TGF-β1, sonicated antigens of *S. pneumoniae* (10 and 50 μg protein/ml) and *K. pneumoniae* (5, 10 and 50 μg protein/ml) reduced the growth of lymphocytes (Table 1). In the absence of IL-6 and TGF-β1, growth of lymphocytes was not inhibited by LPS. However in the presence of IL-6 and TGF-β1, high concentrations (10 and 50 μg protein/ml) of LPS suppressed the multiplication of lymphocytes (Table 1). On the other hand, zymosan A promoted the proliferation of lymphocytes with or without IL-6 and TGF-β1 (Table 1).

**Table 1:** **Effects of microbial antigens on lymphocyte growth with or without IL-6 and TGF**β**1**

**Antigen**	**IL-6(-), TGF-β1(-)**^**a**^		**IL-6(+), TGF-β1(+)**^**a**^				
	**0 μg/ml**	**50 μg/ml**	**0 μg/ml**	**1 μg/ml**	**5 μg/ml**	**10 μg/ml**	**50 μg/ml**
*M. pneumoniae* M129		229.6±19.1^b^		81.9±5.8	101.5±10.9	134.7±15.6^c^	147.8±6.3^c^
*S. pneumoniae* ATCC 33400		18.4±1.2^b^		110.1±6.3	100.9±12.9	66.8±5.2^c^	22.3±2.4^c^
*K. pneumonia* ATCC 13883	111.7±13.0	6.8±4.2^b^	100.0±8.1	109.2±4.1^c^	44.3±1.2^c^	27.3±1.6^c^	6.1±0.7^c^
LPS from *E. coli* 0127: B8		128.8± 6.1^b^		86.5±2.7^c^	89.4±8.1	81.2±5.0^c^	56.5±7.0^c^
Zymosan A from *S. cerevisiae*		197.9±10.2^b^		104.5±10.1	114.8±9.6^c^	124.9±4.0^c^	159.1±5.4

^a^Relative ratio (%) of viable lymphocyte count with or without IL-6 (20 ng/ml) and TGF-β1 (2 ng/ml) stimulated with *M. pneumoniae* and other antigens. Relative ratio is the mean ± standard deviation (four or five samples per group) of the number of viable lymphocytes at day 4.

^b^Significantly different (p < 0.05) from value for cytokine (−), antigen 0 μg/ml by Student’s *t*-test.

^c^Significantly different (p < 0.05) from value for 20 ng/ml of IL-6 and 2 ng/ml of TGF-β1 (+), antigen 0 μg/ml by Dunnett multiple comparison statistical test.

### Effect of *M. pneumoniae* and other antigens on lymphocyte IL-17A production

**Figure 5:**
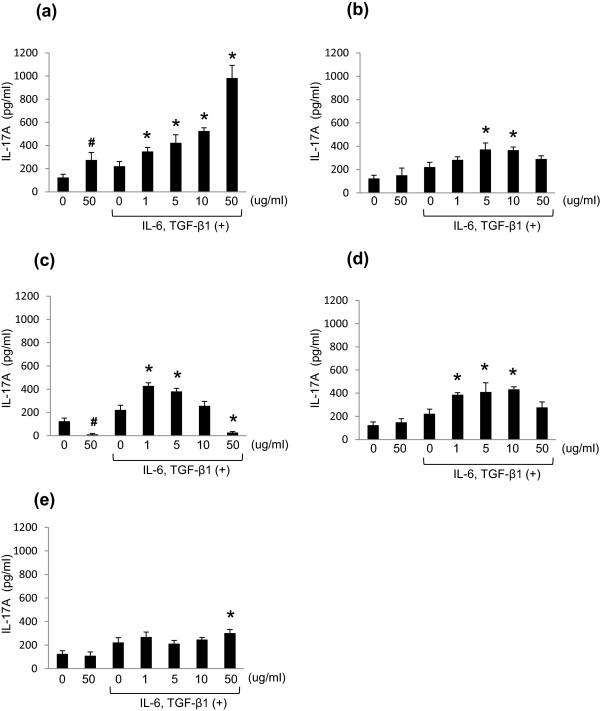
**Effects of *M. pneumoniae *and other antigens on IL-17A production in murine lymphocytes.** IL-17A concentration (pg/ml) in the culture supernatant of murine lymphocytes stimulated with antigens of: *M. pneumoniae* strain M129 **(a)**, *S. pneumoniae* strain ATCC 33400 **(b)**, *K. pneumoniae* strain ATCC 13883 **(c),** LPS from *E. coli* O127:B8 **(d)**, Zymosan A from *S. cerevisiae***(e)**. *p < 0.05 vs. TGF-β1 and IL-6 (+), Ag (−) by Dunnett multiple comparison statistical test; ^#^*p* < 0.05 vs. cytokine (−), Ag (−) by Student’s *t*-test.

*M. pneumoniae* antigens promoted the production of IL-17A. Furthermore, in the presence of IL-6 and TGF-β1, IL-17A production by lymphocytes markedly increased in an antigen concentration-dependent manner (Figure 5a). IL-17A production by lymphocytes induced by either *S. pneumoniae*, *K. pneumoniae* antigens or LPS was increased only twice as much as control in the presence of IL-6 and TGF-β1 (Figure 5b,c,d). The addition of 50 μg protein/ml of *S. pneumoniae* antigens and 50 μg/ml LPS could not induce the levels of IL-17A compared to *M. pneumoniae* antigens (Figure 5b,d). Moreover, very low levels of IL-17A production were observed in the presence of 50 μg protein/ml of *K. pneumoniae* sonicated antigens (Figure 5c) and IL-17A production was not increased by zymosan A stimulation at all (Figure 5e).

### Effect of *M. pneumoniae* and other antigens on lymphocyte IL-10 production

**Figure 6:**
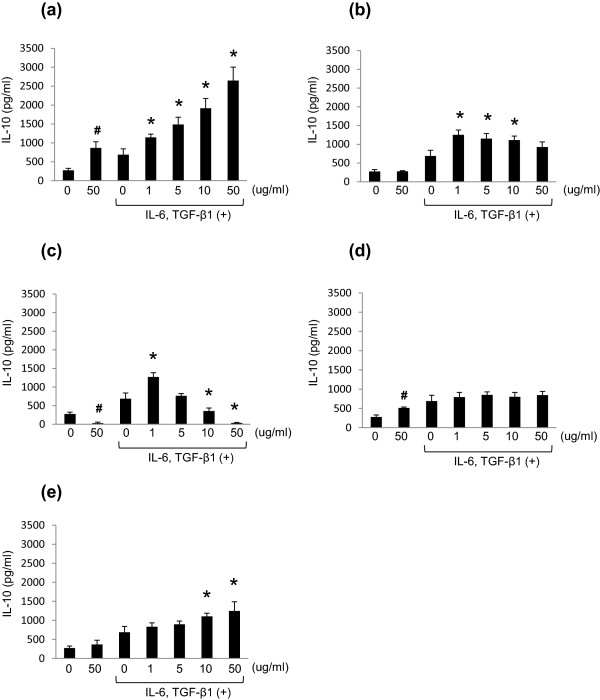
**Effects of *M. pneumoniae *and other antigens on IL-10 production in murine lymphocytes.** IL-10 concentration (pg/ml) in the culture supernatant of murine lymphocytes stimulated with antigens of *M. pneumoniae* strain M129 **(a)**, *S. pneumoniae* strain ATCC 33400 **(b)**, *K. pneumoniae* strain ATCC 13883 **(c)**, LPS from *E. coli* O127:B8, **(d)**, Zymosan A from *S. cerevisiae***(e)**. *p < 0.05 vs. TGF-β1 and IL-6 (+), Ag (−) by Dunnett multiple comparison statistical test; ^#^*p* < 0.05 vs. cytokine (−), Ag (−) by Student’s *t*-test.

*M. pneumoniae* antigens promoted the production of IL-10 (Figure 6a). Furthermore, as for IL-17A, IL-6 and TGF-β1 increased IL-10 production by lymphocytes in an antigen concentration-dependent manner (Figure 6a). IL-10 production by lymphocytes induced by *S. pneumoniae* and *K. pneumoniae* antigens increased only twice as much as control in the presence of IL-6 and TGF-β1 (Figure 6b,c). However, LPS did not induce significant lymphocyte IL-10 production, even in the presence of IL-6 and TGF-β1 (Figure 6d). IL-10 production by zymosan A induction was increased in the presence of IL-6 and TGF-β1, though this was only approximately 50% of that observed in *M. pneumoniae* antigen experiments (Figure 6e).

## Discussion

The pathogenic mechanism by which the diverse extrapulmonary symptoms subsequent to mycoplasma infection occur is thought to be possibly due to indirect tissue injury caused by an overzealous host immune response [11,12].

In this study we investigated the Th17 and Treg based immune response to mycoplasmal diseases using IL-17A and IL-10 as index markers. It was therefore suggested that extrapulmonary complications subsequent to the development of mycoplasmal pneumonia were due to breakdown of the immune response.

Histological inflammation was induced in the murine lung by nasal inoculation of *M. pneumoniae* antigens, and the levels of inflammation correlated with sensitization conditions in this in vivo study. Severe inflammation was observed in the higher-dose and frequent sensitization group (Group A). Moreover, mRNA expression of TNF-α and KC proinflammatory cytokines supported the histopathological findings. This in vivo analysis revealed that *M. pneumoniae* antigens were also capable of inducing chemokines in our antigen induced inflammation model. Intrapulmonary concentrations of IL-17A in BALB/c mice were increased in Group A and B which were sensitized frequently or sensitized with higher amounts of *M. pneumoniae* antigens. We inferred that the positive effector T cell balance (Th1-Th2-Th17) of the antigen induced inflammation model was a persistent Th17 dominant condition, as intrapulmonary Th1 and Th2 cytokines IFN-γ and IL-4 were not detected but high concentrations of IL-17A and high expression levels of IL-17A mRNA were detected in the lung of BALB/c mice. The immunological response causes migration and generation of neutrophils, which plays a part not only in host defense from bacterial infection but also as a pathological mechanism for autoimmune diseases such as chronic rheumatoid arthritis [27,28]. Our experimental results demonstrated that even repetitive sensitization with a small amount of *M. pneumoniae* antigens induced a Th17 dominant immune response. This discovery raises the possibility that clinically mild symptoms observed in mycoplasmal pneumonia caused by a small bacterial colonization load may still result in enhancement of the Th17 response, eliciting host autoimmune diseases by persistent infection. Therefore, it is not only simple infection but the antigen inoculation conditions that are involved in the onset of extrapulmonary complications resembling autoimmune disease.

It was recently reported that polysaccharide derived from *Bacteroides fragilis* activated Treg cells and promoted a production of IL-10 in the intestinal tract [29]. Both factors elevate the intrapulmonary concentration of IL-10 and up regulate IL-10 mRNA expression in the lungs of BALB/c mice representing persistent IL-10 production in this *M. pneumoniae* antigen induced inflammation model.

It was previously reported that IL-10 deficient mice developed spontaneous enterocolitis similar to human inflammatory bowel disease [30], and it was proven that large quantities of IL-10 improved formalin or dextran sulfate sodium (DSS) induced colitis [31,32]. We therefore suspected that IL-10 was produced in our antigen induced inflammation model as demonstrated previously. Thus when IL-10 production is decreased by inhibition of Tr1 differentiation, lung inflammation induced by *M. pneumoniae* antigens cannot be mitigated, and extrapulmonary complications similar to autoimmune diseases may also occur in vivo. These results suggest that inflammation induced by IL-17A production in Th17 cells is mitigated by Tr1 production of IL-10.

We could not confirm the inhibitory effect of Th3 cells on immune responses at inflammatory sites, as TGF-β1 mRNA expression did not correlate with the frequency of sensitization or dose in this antigen induced inflammation model.

CD4^+^CD25^+^T cells express cytotoxic T-lymphocyte antigen 4 (CTLA-4) with membrane-associated TGF-β on the cell surface, which suppresses multiplication of positive effector T cells by direct cytoadherence [33,34]. Foxp3, a master regulatory gene is constitutively expressed in CD4^+^CD25^+^T cells [35], and both Tr1 and Th3 cells are negative for Foxp3 [36,37]. It was assumed that intrapulmonary Foxp3 mRNA expression is not increased as drastically in comparison with IL-10, as frequent and large quantity sensitization with *M. pneumoniae* antigens induced CD4^+^CD25^+^T cell translocation from thymus to the lung.

Additionally, we performed an in vitro analysis aimed to evaluate the specificity of immuno-inducibility and Th17-differentiation enhancability of *M. pneumoniae* antigens. It was reported that IL-6 and TGF-β1 are necessary for early differentiation of the Th17 cell from naïve T cells [38]. Therefore, mouse lymphocytes were primed with *M. pneumoniae* antigens in the presence of IL-6 and TGF-β1. Furthermore, in order to simulate the presentation of *M. pneumoniae* antigens by dendritic cells in vitro, we added anti-CD3 antibodies and anti-CD28 antibodies.

Compared to saline control, 50 μg protein/ml of *M. pneumoniae* antigen stimulation significantly induced IL-17A production by mouse lymphocytes from day 2 to 5, with greater than sixfold production observed on day 3 (Figure 4a). Additionally, IL-10 production showed a significant increase from day 1 to 5 (Figure 4b). This showed that IL-17A and IL-10 production in vitro induced by *M. pneumoniae* antigen sensitization mirrored the in vivo antigen induced inflammation model.

When we compared viable cell count at the peak of IL-17A and IL-10 production on day 4, 50 μg protein/ml of *M. pneumoniae* antigens induced multiplication of mouse lymphocytes approximately twofold compared to saline control. Though mildly increased growth rates were observed in the presence of IL-6 and TGF-β1, higher concentrations of *M. pneumoniae* antigens induced activation and proliferation of lymphocytes (Table 1). IL-17A and IL-10 production were enhanced in a concentration-dependent manner by *M. pneumoniae* antigens, and the presence of IL-6 and TGF-β1 led to further production of IL-17A and IL-10 (Figures 5a, 6a), showing induction of the two genes under a Th17 dominant immune balance both in vivo and in vitro.

With respect to the effects of antigens prepared from bacteria causing a classical pneumonia, 50 μg protein/ml of *S. pneumoniae* sonicated antigens imposed a lethal effect on lymphocytes, with decreased viability to 18% of saline control, possibly through the effect of pneumolysin (Table 1). *S. pneumoniae* is well known to produce various virulence factors, and pneumolysin is an intracellular cytotoxin causing lysis of the cytoplasmic membrane of host cells by perforation [39]. Due to lymphocyte death and reduction of activity by pneumolysin containing *S. pneumoniae* sonicated antigens, IL-17A and IL-10 production was not observed in a concentration-dependent manner (Figures 5b, 6b).

Regardless of the addition of IL-6 and TGF-β1, 50 μg protein/ml of *K. pneumoniae* antigens had a distinct lethal effect on mouse lymphocytes, with a viability of approximately 6% at 4 days. Cell death in this experiment was observed in a *K. pneumoniae* antigen concentration-dependent manner (Table 1). Recently, it was reported that *K. pneumoniae* produced various lethal active metabolites including cytotoxins, hydrolytic enzymes and haemolysins similar to *S. pneumoniae*[40,41] and both IL-17A and IL-10 production were decreased as expected by exposure to *K. pneumoniae* antigens (Figures 5c, 6c).

The difference in pathogenic mechanism between *M. pneumoniae* and other pulmonary pathogenic bacteria can be explained by the results of in vitro analyses. The antigens derived from bacteria causing pneumonia showed lethality to immunocytes, but *M. pneumoniae* antigens lead to activation of host immune responses.

LPS is recognized by TLR 4 and activates macrophages [42,43]. However, in this in vitro study, LPS did not induce proliferation of lymphocytes (Table 1). In addition, LPS stimulated IL-17A and IL-10 production did not occur in a concentration-dependent manner (Figures 5d, 6d). It was considered that in comparison with *M. pneumoniae* antigen, LPS has minimal effect on Th17 cell differentiation.

Zymosan A is recognized by a polymer of TLR2 and TLR1 or TLR6, causing macrophage activation [44]. Zymosan A induced proliferation of lymphocytes and IL-10 production in a concentration-dependent manner similar to *M. pneumoniae* antigens (Table 1, Figure 6e). However, there was no significant dose-dependant increase in IL-17A production (Figure 5e) and so we did not consider Zymosan A to be a major player in Th17 cell differentiation. Zymosan A induces not only innate immunity but a Th17 response via Jagged1 activation on the dendritic cell and was recently reported as a Th17 adjuvant [45]. From the above, we can conclude that Zymosan A alone without other immune cells can activate the proliferation of lymphocytes, but cannot induce a potent Th17 response even in the presence of IL-6 and TGF-β1.

## Conclusions

In this study, it was shown that *M. pneumoniae* antigens induced potent immunoreaction and enhanced the Th17 cell response both in vivo and in vitro, and that both Treg and IL-10 are involved in the suppression of IL-17A production. This raises the possibility that breakdown of the immune balance may be part of the process leading to subsequent development of extrapulmonary mycoplasmal pneumonia.

## Competing interests

The authors declare no competing interests concerning this work.

## Authors’ contributions

SKu and SKa conceived and designed the experiments. SKu and TO performed animal experiments. SKu and HY performed real time PCR procedures. SKu, SKa and HT analyzed the data. TO, HY and KA contributed reagents/materials/analysis tools. All authors read and approved the final manuscript.
